# FIND Tuberculosis Strain Bank: a Resource for Researchers and Developers Working on Tests To Detect Mycobacterium tuberculosis and Related Drug Resistance

**DOI:** 10.1128/JCM.01662-16

**Published:** 2017-03-24

**Authors:** Belay Tessema, Pamela Nabeta, Eloise Valli, Audrey Albertini, Jimena Collantes, Nguyen Huu Lan, Elena Romancenco, Nestani Tukavdze, Claudia M. Denkinger, David L. Dolinger

**Affiliations:** a Foundation for Innovative New Diagnostics, Geneva, Switzerland; b Department of Medical Microbiology, University of Gondar, Gondar, Ethiopia; c Universidad Peruana Cayetano Heredia, Lima, Peru; d Pham Ngoc Thach Hospital, Ho Chi Minh City, Vietnam; e National Reference TB Laboratory, Institute of Phthisiopneumology, Chisinau, Moldova; f National Center for Tuberculosis and Lung Disease, Tbilisi, Georgia; Carter BloodCare and Baylor University Medical Center

**Keywords:** strain bank, drug-resistant tuberculosis, diagnostics development

## Abstract

The spread of multidrug-resistant (MDR) tuberculosis (TB) and extensively drug-resistant (XDR) TB hampers global efforts in the fight against tuberculosis. To enhance the development and evaluation of diagnostic tests quickly and efficiently, well-characterized strains and samples from drug-resistant tuberculosis patients are necessary. In this project, the Foundation for Innovative New Diagnostics (FIND) has focused on the collection, characterization, and storage of such well-characterized reference materials and making them available to researchers and developers. The collection is being conducted at multiple centers in Southeast Asia, South America, Eastern Europe, and soon the sub-Saharan Africa regions. Strains are characterized for their phenotypic resistances and MICs to first-line drugs (FLDs) and second-line drugs (SLDs) using the automated MGIT 960 system following validated procedures and WHO criteria. Analysis of resistance-associated mutations is done by whole-genome sequencing (WGS) using the Illumina NextSeq system. Mycobacterial interspersed repetitive-unit–variable-number tandem-repeat analysis and WGS are used to determine strain lineages. All strains are maintained frozen at −80°C ± 10°C as distinct mother and daughter lots. All strains are extensively quality assured. The data presented here represent an analysis of the initial part of the collection. Currently, the bank contains 118 unique strains with extracted genomic DNA and matched sputum, serum, and plasma samples and will be expanded to a minimum of 1,000 unique strains over the next 3 years. Analysis of the current strains by phenotypic resistance testing shows 102 (86.4%), 10 (8.5%), and 6 (5.1%) MDR, XDR, and mono/poly resistant strains, respectively. Two of the strains are resistant to all 11 drugs that were phenotypically tested. WGS mutation analysis revealed FLD resistance-associated mutations in the *rpoB*, *katG*, *inhA*, *embB*, *embA*, and *pncA* genes; SLD resistance in the *gyrA*, *gyrB*, *rrs*, *eis*, and *tlyA* genes; and ethionamide resistance in the *ethA* genes. Most important lineages are represented in the bank, and further collections have been initiated to increase geographic and lineage diversity. The bank provides highly characterized and high-quality strains as a resource for researchers and developers in support of the development and evaluation of new diagnostics and drug resistance detection tools.

## INTRODUCTION

Drug-resistant tuberculosis (DR-TB) threatens global TB control efforts. Worldwide, 3.3% of new TB patients and 20% of previously treated patients were estimated to have multidrug-resistant tuberculosis (MDR-TB). While extensively drug-resistant TB (XDR-TB), defined as MDR-TB plus resistance to at least one fluoroquinolone (FQ) and a second-line injectable drug (SLID), has been reported by 105 countries by the end of 2014 (1), only 26% of the estimated MDR-TB cases were detected in 2014 (1). Accurate diagnosis of TB and rapid detection of drug resistance are critical to the timely initiation of an effective treatment. Without rapid identification of MDR-TB, the ambitious “End TB” goals of the World Health Organization (WHO) will not be achievable (2).

Molecular methods for drug susceptibility testing (DST) have considerable advantages when programmatic management of DR-TB is scaled up, particularly with regard to time-to-result and also with standardization and reduced requirements for biosafety (3, 4). However, our understanding of the resistance mechanisms for some first-line drugs (FLDs) and second-line drugs (SLDs) is still limited. For the majority of drugs, multiple genes are involved, mutations may be scattered over long gene sequences, and a percentage of resistance-associated genomic markers or mechanisms may not have been identified. These factors complicate the development of new rapid molecular tests to detect resistance in the Mycobacterium tuberculosis complex (MTC) (5, 6).

To support the research and development of new diagnostic assays and solutions, a better understanding of the genotypic basis of resistance is essential and highly characterized wild-type and resistant strains need to be made available to developers. A better understanding of the genotypic basis of resistance can only be achieved through large databases and data sets that correlate phenotypic and genotypic resistance characterization, such as the ReSeqTB initiative of FIND, Critical Path (C-Path), United States Centers for Disease Control and Prevention (CDC), WHO, and the Bill and Melinda Gates Foundation (BMGF) (7). Existing strain banks, such as the Special Programme for Research and Training in Tropical Diseases (TDR), in collaboration with the Institute of Tropical Medicine (ITM), Antwerp, Belgium, have made M. tuberculosis isolates available upon request (8, 9). However, a wider diversity of genetically and clinically well-characterized resistant strains is required, particularly when it comes to second-line drugs.

To fill this gap, FIND has established a strain bank that is complementary to the existing banks by focusing more specifically on MDR, pre-XDR, and XDR strains with extensive lineage backgrounds. Herein, we describe the current collection of strains within the FIND TB strain bank, the characterization of strains, the nature of services provided to researchers and developers, and future plans.

## RESULTS

At the time of this analysis, the FIND TB strain bank contains 118 well-characterized strains with matched daughter aliquots, DNA extracts, sputum samples, EDTA plasma, and serum samples.

### Clinical and geographical distribution of the strains.

Based on the results of phenotypic DST, 102 (86.4%), 10 (8.5%), and 6 (5.1%) of the strains are classified as MDR, XDR, or mono/poly resistant strains, respectively. The current regional composition of the strains shows that 49.2% of the strains are from Southeast Asia, 37.3% are from South America, and 13.6% of the strains are from Eastern Europe. Currently, the majority of the strains are from relapse cases or treatment failures (28.0% and 27.1%, respectively). Six (5.1%) of the strains are from tuberculosis patients with a comorbidity of HIV infection (Table 1).

**TABLE 1:** Clinical and demographic characteristics of MDR- and XDR-TB patients^*a*^

Characteristic	MDR-TB		XDR-TB		Other^*b*^		Total	
	No.	%	No.	%	No.	%	No.	%
Region of origin								
Southeast Asia	53	52.0	0	0.0	5	83.3	58	49.2
South America	41	40.2	2	20.0	1	16.7	44	37.3
Eastern Europe	8	7.8	8	80.0	0	0.0	16	13.6
Subtotal	102	100.0	10	100.0	6	100.0	118	100.0
History of previous treatment								
Relapse	30	29.4	3	30.0	0	0.0	33	28.0
Failure	24	23.5	3	30.0	5	83.3	32	27.1
New	22	21.6	3	30.0	1	16.7	26	22.0
Defaulter	7	6.9	0	0.0	0	0.0	7	5.9
Unknown	19	18.6	1	10.00	0	0.0	20	17.0
Subtotal	102	100.0	10	100.0	6	100.0	118	100.0
HIV status								
Positive	6	5.9	0	0.0	0	0.0	6	5.1
Negative	87	85.3	9	90.0	6	100.0	102	86.4
Unknown	9	8.8	1	10.0	0	0.0	10	8.5
Subtotal	102	100.0	10	100.00	6	100.0	118	100.0
M. tuberculosis lineages								
Beijing	59	57.84	4	40.0	5	83.3	68	57.6
LAM^*c*^	27	26.47	1	10.0	1	16.7	29	24.6
X-type	6	5.88	1	10.0	0	0.0	7	5.9
URAL	1	0.98	4	40.0	0	0.0	5	4.2
Haarlem	4	3.92	0	0.0	0	0.0	4	3.4
Clade 1	4	3.92	0	0.0	0	0.0	4	3.4
Delhi/CAS	1	0.98	0	0.0	0	0.0	1	0.9
Subtotal	102	100.00	10	100.0	6	100.0	118	100.0

a There were a total of 118 patients.

b Other indicates monoresistant or polyresistant M. tuberculosis strains.

c LAM, Latin America-Mediterranean.

### Genetic diversity of the strains.

The lineage diversity of the strains based on mycobacterial interspersed repetitive-unit–variable-number tandem-repeat (MIRU-VNTR) typing and whole-genome sequencing (WGS) results is Beijing (57.6%), LAM (24.6%), X-type (5.9%), URAL (4.2%), Haarlem (3.4%), clade 1 (3.4%), and Delhi/Central Asian Strain (CAS) (0.9%). The majority of MDR-TB strains are of Beijing (57.8%) and of Latin American Mediterranean (LAM) (26.5%) origins. While the majority of the XDR-TB strains are of Beijing (40.0%) and URAL (40.0%) origins. The Beijing lineage is the predominant lineage among strains collected from Southeast Asia (55; 94.8%) and Eastern Europe (12; 75.0%), while LAM is the predominant lineage among strains from South America (29; 65.9%) (Table 1; see also Table S1 in the supplemental material).

### First-line anti-TB drug resistance.

**FIG 1:**
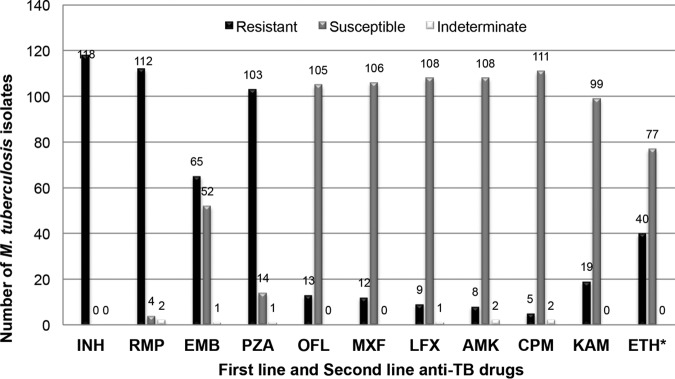
Drug resistance profile of 118 M. tuberculosis isolates based on phenotypically DST results. INH, isoniazid; RMP, rifampin; EMB, ethambutol; PZA, pyrazinamide; OFL, ofloxacin; MXF, moxifloxacin; LFX, levofloxacin; AMK, amikacin; CPM, capreomycin; KAM, kanamycin; ETH, ethionamide. *, one isolate was contaminated during DST for ETH.

First-line drug resistance profiles of the initial 118 strains based on phenotypic DST is indicated in Fig. 1. All strains are resistant to isoniazid (INH), while 94.9%, 55.1%, and 87.3% of the strains are resistant to rifampin (RMP), ethambutol (EMB), and pyrazinamide (PZA), respectively. Four strains have indeterminate results, 2 for RMP, 1 for EMB, and 1 for PZA. Approximately half (48.3%) of the strains are resistant to all of the first-line drugs (FLDs) (INH, RMP, EMB, PZA) tested (see Table S2 in the supplemental material). Forty strains (33.9%) are resistant to INH, RMP, and PZA, and 6 strains (5.1%) are resistant only to INH and RMP.

### Second-line anti-TB drug resistance.

Second-line drug resistance profiles of the initial 118 strains based on phenotypic DST results are also summarized in Fig. 1. The highest proportion of resistance is observed for ethionamide (ETH) (34.2%) followed by kanamycin (KAM) (16.1%), ofloxacin (OFL) (11.0%), moxifloxacin (MXF) (10.2%), levofloxacin (LFX) (7.6%), amikacin (AMK) (6.8%), and capreomycin (CPM) (4.2%). Five strains have indeterminate result to second-line drugs, one strain to LFX, two strains to AMK, and two strains to CPM. A large proportion of the strains (39.8%) are resistant to at least 1 second-line drug. The highest proportion of mono-resistance was observed to ETH (22.1%) (see Table S3 in the supplemental material).

### Frequency of mutations conferring resistance to FLDs.

Of the total strains analyzed by WGS, 92.4% have *rpoB* gene mutations conferring resistance to RMP while the remaining 7.6% that have been shown to be phenotypically resistant showed no mutations within the *rpoB* gene. The most frequent mutation observed in the *rpoB* gene is S450L (62.7%) followed by D435V (10.2%). Four phenotypically RMP-susceptible strains have been shown to have *rpoB* gene mutations: L452P (2 strains), S450L (1 strain), and L430P (1 strain) mutations. Additional data are provided in Table S4 in the supplemental material.

All phenotypic INH-resistant strains have resistance-conferring mutations either in the *katG* or the *inhA* gene. The majority of strains (89.8%) have mutations in the *katG* gene, with the highest frequency being the S315T mutation (86.4%). The most common *inhA* gene mutation is −15C/T (10.2%).

All phenotypic EMB-resistant strains (65) have resistance-associated mutations in *embB* (92.3%), *embA* (3.1%), or in both the *embB* and *embA* (4.6%) genes. The most frequent mutation among EMB-resistant strains is the M306V mutation (36.9%) followed by the M306I mutation (20.0%) in the *embB* gene.

Among PZA-resistant strains, 81.9% had mutations in the *pncA* gene while the remaining PZA-resistant strains (18.1%) had no identified mutations in the *pncA* gene. H51R, Q10R, and −11A/G are the most frequent mutations observed (6.7% each) in the *pncA* gene among phenotypic PZA-resistant strains. Analysis of the phenotypically PZA-susceptible strains showed that 58.3% (7/12) of the strains had resistance-associated mutations in the *pncA* gene.

### Frequency of mutations conferring resistance to SLDs.

Of 14 phenotypically resistant strains to at least one of the fluoroquinolone (FQ) drugs tested, 13 strains had a FQ resistance-associated mutation in the *gyrA* gene, while one phenotypic FQ-resistant strain had an E501D mutation in the *gyrB* gene. The most frequent mutation among phenotypic FQ-resistant strains was D94G (6 strains) followed by D94A (4 strains). Additional data are provided in Table S5 in the supplemental material.

Among the 19 observed strains that were phenotypically resistant to SLIDs, 4 strains had mutations in the *rrs* gene, 8 strains had mutations in the *eis* gene, one had mutations in the *tlyA* gene; the remaining 6 strains had unidentified mechanisms for resistance. The most frequently observed mutations among phenotypic SLID-resistant strains is the 1401A/G (3 strains) mutation in the *rrs* gene and −12C/T (4 strains) mutation in the *eis* gene.

Of 40 phenotypic ETH-resistant strains, 23 strains had mutations associated with ETH in the *ethA* gene. The remaining 17 phenotypic ETH-resistant strains had unidentified mechanisms for resistance. The most frequent ETH resistance-associated mutation observed was the −15C/T (17/23 strains) mutation in the *ethA* gene.

## DISCUSSION

### Current status of the strain bank.

The FIND TB strain bank represents a unique resource for highly characterized and high-quality MDR-TB and XDR-TB strains that is complementary to other existing banks (8, 9). It is comprised of well-characterized and quality assured strains. The bank includes strains from geographically diverse populations and diverse phylogenetic lineages. In addition to the strains, the bank contains matched DNA extracts and matched clinical specimens: sputum samples, serum samples, and plasma. To achieve the initial target of 1,000 characterized strains by early 2018 and to increase geographic coverage and strain lineage diversity, FIND is adding collection sites in South Africa (Kwazulu Natal and Cape Town) and Vietnam. The main purpose of this bank is to facilitate access to quality assured reference materials and thereby to stimulate and enhance the development and validation of innovative, novel diagnostic and drug resistance detection tools that are fit-for-purpose for limited resource settings (10). Other strains relevant for test development are also being added (e.g., nontuberculous mycobacteria to support exclusivity testing).

The bank has attempted to follow best practices during collection, characterization, and storage of the reference materials (11). The viability, purity, and authenticity of strains are carefully checked before distribution.

### Access to the strain bank.

Strains, DNA extracts, and matched clinical specimens from this collection are available upon request from FIND. A Strain Bank Review Committee (SBRC), composed of five qualified members, reviews all applications.

Requests addressing the following criteria are given the highest priority: (i) relevance to public health (feasibility and impact on patients and disease control programs), (ii) applicability of the technology in high-burden countries as predefined in target product profiles (10, 12), (iii) estimated low cost of the final product, and (iv) previous data or scientific evidence supporting the request. All applicants are also required to sign a material transfer agreement (MTA) regulating the use of the materials. The MTA and other necessary information to request reference materials are available on the FIND website (http://www.finddx.org/specimen-banks/material-request-form/).

### Virtual strain bank.

Key information pertaining to the FIND strains is portrayed in the FIND virtual strain bank. In addition, the FIND virtual strain bank will also provide a portal in which other researchers, who house well-characterized strains, can make key characteristics of their strains visible to researchers and developers. The online portal will be searchable and will allow researchers and developers to determine who might have a strain of interest. The researcher or developer can then contact the owner of the particular strain (directly or through FIND) to obtain access to the strain. As such, the portal will further facilitate collaboration among TB researchers and developers.

### Conclusion.

The FIND TB strain bank is an inimitable resource that encourages researchers and developers and accelerates the development of novel, affordable TB diagnostics for the detection of drug resistance in TB.

## MATERIALS AND METHODS

### Study design and patient recruitment.

A longitudinal, multicenter study is being conducted to collect and store reference materials of adult patients who at least have DR-TB. Patients presumed to have MDR-TB have been recruited at sites in South America (Peru), Southeast Asia (Vietnam), and Eastern Europe (Moldova and Georgia) since August 2014. The initial enrollment target for this project has been set at 1,000 patients confirmed to have DR-TB. Patients presumed to have DR-TB are defined as TB patients who are either (i) new TB suspects with rifampin (RMP) resistance indicated by the GeneXpert MTB/RIF assay (Xpert MTB/RIF; Cepheid, Sunnyvale, CA, USA), (ii) TB relapse cases, (iii) retreatment cases after default, (iv) failure of category I or II treatment regimen, or (v) contacts with MDR-TB cases. All patients recruited for an in-depth characterization of strains at reference laboratories must at least have confirmed RMP resistance as determined by Xpert MTB/RIF.

Written informed consent is obtained from all eligible study participants prior to data and sample collection. Patients with presumed MDR-TB who have only extrapulmonary TB, are less than 18 years of age, or are unable to provide informed consent are excluded from the study. Study participants are provided the standard anti-TB treatment regimens based on national treatment guidelines. This study has been approved by the appropriate ethical review board of FIND and the boards of the collection sites.

### Sample collection and processing.

Clinical and demographic information of patients with presumed DR-TB is collected using a standardized questionnaire. Three sputum samples, spot-morning-spot (≥3 ml each), and 20 ml of blood (10 ml for EDTA plasma and 10 ml for serum) are collected from all eligible patients prior to the start of treatment. On site, the first sputum samples are processed and characterized by smear microscopy, Xpert MTB/RIF, and culture using Lowenstein-Jensen (LJ) medium and the Bactec mycobacterial growth indicator tube (MGIT) 960 system (Becton Dickinson [BD], Sparks, MD, USA). Species identification is performed using either the BD MGIT TBc identification test, Capilia TB-Neo (Tauns laboratories, Japan), or TB Ag MPT64 Rapid test (Standard Diagnostics, South Korea).

**FIG 2:**
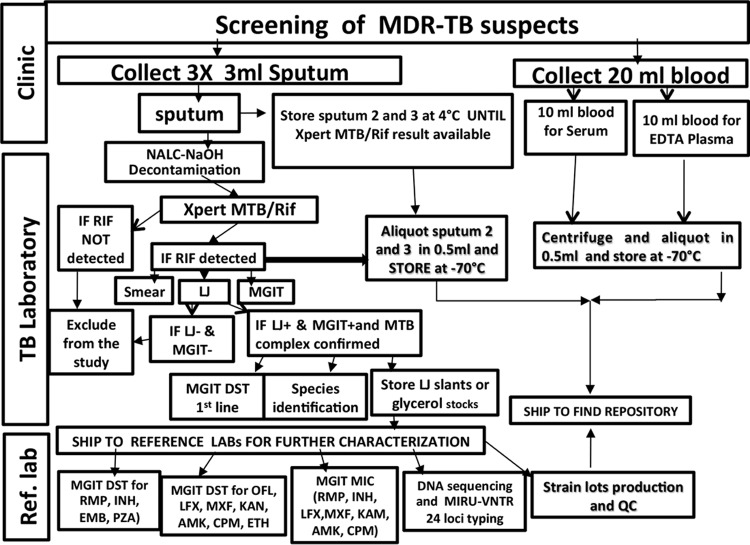
Multidrug-resistant TB suspect identification, specimen collection, storage, transportation, and strain characterization. QC, quality controls; INH, isoniazid; RMP, rifampin; EMB, ethambutol; PZA, pyrazinamide; OFL, ofloxacin; MXF, moxifloxacin; LFX, levofloxacin; AMK, amikacin; CPM, capreomycin; KAM, kanamycin; ETH, ethionamide.

The remaining sputum samples (sputum sample 2 and sputum sample 3) are homogenized with glass beads, aliquoted in 0.5-ml volumes, and stored at −80 ± 10°C as are EDTA plasma and serum until shipped to the FIND repository partner. HIV testing is also done following the appropriate national HIV testing algorithm at each site. Aliquoted sputum, plasma, and serum samples are shipped to the FIND repository at ZeptoMetrix (Franklin, MA, USA) for storage and distribution to end users upon request. LJ slants or glycerol stocks of culture isolates from positive cultures are shipped to the National Reference Center for Mycobacteria (Borstel, Germany) or the National Jewish Health (Denver, CO, USA) TB reference laboratories for in-depth characterization of the isolated strains (Fig. 2).

### Strain characterization.

All LJ slants sent to the reference laboratories for in-depth characterization are subcultured on LJ medium. A single dominant colony is picked from every culture and subsequently propagated on LJ medium. All subsequent tests are carried out from these single-colony-derived cultures.

### Phenotypic drug susceptibility testing.

Phenotypic DST for first-line drugs (FLDs) and second-line drugs (SLDs) is done using the WHO-endorsed proportion method on the MGIT 960 system. DST is done for the following FLDs: isoniazid (INH), rifampin (RMP), ethambutol (EMB), and pyrazinamide (PZA), and for the following SLDs: ofloxacin (OFL), moxifloxacin (MXF), levofloxacin (LFX), amikacin (AMK), capreomycin (CPM), kanamycin (KAM), and ethionamide (ETH). The critical concentrations used are in accordance with WHO recommendations (13): INH (0.1 μg/ml), RMP (1 μg/ml), EMB (5 μg/ml), PZA (100 μg/ml), OFL (2 μg/ml), MXF (0.5 μg/ml), LFX (1.5 μg/ml), AMK(1 μg/ml), KAM (2.5 μg/ml), CPM (2.5 μg/ml), and ETH (5 μg/ml). The data are evaluated when the control reaches 400 growth units. A strain is termed resistant when the growth unit of the drug tube is 100 or more. A strain is termed susceptible if the growth unit of the drug tube is 0 growth units, and a strain is termed indeterminate if growth units range from 1 to 99. As part of the phenotypic DST, MICs are also determined using the MGIT 960 system for the following drugs and concentrations in micrograms per milliliter: RMP (1, 4, 20), INH (0.1, 0.4, 1, 3, 10), LFX (0.1875, 0.375, 0.75, 1.5, 3,7.5, 15), MXF (0.0625, 0.125, 0.25, 0.5, 1, 2.5, 7.5), KAM (0.3125, 0.625, 1.25, 2.5, 5, 12.5, 25), AMK (0.125, 0.25, 0.5, 1, 4, 20, 40), and CPM (0.3125, 0.625, 1.25, 2.5, 5, 12.5, 25) (14, 15).

### Whole-genome sequencing-based drug susceptibility analysis.

Whole-genome sequencing (WGS) is performed using the Illumina NextSeq (Illumina, San Diego, CA, USA) system. The drug resistance-determining regions (RDRs) of the following genes: *rpoB* (RMP), *katG* and *inhA* (INH), *embB* and *embA* (EMB), *pncA* (PZA), *gyrA* and *gyrB* (OFX, MXF, and LFX), *rrs* (KAM, CPM, and AMK), *eis* (KAM), *tlyA* (CPM), and *ethA* (ETH), and the promoters of *inhA* and *eis* are then systematically analyzed for mutations associated with drug resistance based on the recently published database (16). All sequences of the strains are deposited in the ReSeqTB data sharing platform.

### Genotyping.

All strains are typed in Borstel using mycobacterial interspersed repetitive-unit–variable-number tandem-repeat (MIRU-VNTR) 24 loci protocol and by analysis of WGS data to determine phylogenetic lineages (17–19).

### Strain preservation.

As the FIND strain bank is designed to be a long-term resource, storage of the strains in a manner that ensures their viability is essential. Therefore, all strains are stored as mother and daughter lots. Mother lots are divided into two specific lots. One set of mother lots is saved in a separate location for longer term storage and recovery purposes. The other set of mother lots is utilized to replenish daughter lots. Daughter lots are distributed to end users upon request. Strain lot production is performed by the Institute of Microbiology and Laboratory Medicine (IML) (Gauting, Germany).

### Mother lots.

To ensure long-term storage of the strains, mother lots are produced as follows: 1.0 ml of the primary strain stock is used to inoculate one MGIT with Middlebrook 7H9 medium supplemented with oleic acid-albumin-dextrose-catalase (OADC) enrichment (BD) and polymyxin B, amphotericin B, nalidixic acid, trimethoprim, aziocillin (PANTA) antibiotic mixture (BD). A total of 2 MGIT tubes are inoculated and incubated for each strain according to standard MGIT protocol. MTC cells are harvested from the culture by centrifugation for 5 min at 3,500 × *g* (Sigma 3K-15). The concentration of cells is assessed by measuring the adjusted optical density at 580 nm (AOD_580_). An *A*_580_ of 0.1 units is equivalent to approximately 6.3 × 10^7^ CFU mycobacterium cells/ml (20).

Based on the AOD_580_ result, the cells are aseptically diluted to a concentration of approximately 5 × 10^6^ CFU/ml with sterile supplemented 7H9 media. Then, sterile supplemented 7H9 medium containing 20% glycerol is slowly added to the suspension until the final concentration of the strain is approximately 1 × 10^6^ CFU/ml and the final concentration of glycerol is 4% (vol/vol). Finally, the solution (1.0 ml) is aliquoted into sterile cryovials (up to 10 mother aliquots are obtained per MGIT tube). These aliquots are labeled as a specific mother lot. After inspection of the dispensed mother aliquots, the cryovials are placed in a cryobox and stored at −80 ± 10°C and shipped to the FIND repository.

### Daughter lots.

Daughter lots, which are used for distribution to end users, are produced from a mother lot aliquot as follows: 0.5 ml of the mother aliquot is removed aseptically and used to inoculate new MGIT tubes (7H9 supplemented with OADC and PANTA). A total of 2 MGIT tubes are inoculated and incubated for each daughter lot. Cells are harvested by centrifugation for 5 min at 3,500 × *g* (Sigma 3K-15). Cell concentration is determined by obtaining the optical density at 580 nm as previously stated. Based on the optical density result, the cells are aseptically diluted to a concentration of approximately 1 × 10^6^ CFU/ml with sterile supplemented 7H9 medium. The resulting cell suspension is then dispensed in 0.5-ml aliquots into sterile appropriately labeled cryovials as a specific daughter lot aliquot (up to 15 daughter lots are obtained from one MGIT tube). The cryovials are placed in a cryobox and stored at −80 ± 10°C until shipped to the FIND repository.

### Genomic DNA extraction.

Five daughter lot aliquots are pooled, extracted, and purified for DNA using the Chemagic Star DNA Blood400 kit (PerkinElmer, Waltham, MA, USA). The resulting extract from the procedure is quantified by spectrophotometry, and the estimated quantity of DNA is documented. Once extraction and purification are done, the resulting extract is equally split into 5 appropriately labeled vials, placed in a cryobox, stored at −80 ± 10°C, and shipped to the FIND repository.

### Quality control testing of daughter lots.

Upon production of daughter lots, the daughter lots are tested for purity, viability, and authenticity.

### (i) Purity.

Purity/contamination (no bacteria other than MTC) testing is done by inoculating 0.1 ml from a daughter lot aliquot on a blood agar plate. This is done from 1 aliquot and on three separate blood agar plates; plates are incubated at 37°C, 42°C, and at room temperature for 72 h and monitored every 24 h for contaminant growth.

### (ii) Viability.

Viability (growth in culture) testing is done using one aliquot (0.5 ml) from the daughter production, which is inoculated into an MGIT culture tube (7H9 supplemented with OADC and PANTA) for growth of MTC. A positive result is defined by growth within 14 days of incubation. The time to positivity is checked and compared with the time to positivity observed during the production of the daughter lot.

### (iii) Authenticity.

Authenticity (MIRU typing to confirm strain) testing is done using the extracted DNA from each daughter lot production by loci-specific sequencing. Recipient sites are also encouraged to run their own susceptibility testing on the strains in parallel with any molecular tests to minimize the impact of inadvertent variant selection that may not be detected by authenticity testing.

A total of 5 daughter lot vials are used for quality assurance testing of the total lots (30 daughter lots) of each strain. When daughter lots pass the three quality checks, the lots are shipped for storage and distribution to end users upon request.

## Supplementary Material

Supplemental material
