# Supplementary material for: FIND Tuberculosis Strain Bank: a Resource for Researchers and Developers Working on Tests To Detect Mycobacterium tuberculosis and Related Drug Resistance
Source: J Clin Microbiol. 2017 Mar 24;55(4):1066–73. doi: 10.1128/JCM.01662-16 (PMC5377833; doi:10.1128/JCM.01662-16)
Supplement: Supplemental material [file supp_55_4_1066__index.html]

Supplemental material 

# FIND Tuberculosis Strain Bank: a Resource for Researchers and Developers Working on Tests To Detect Mycobacterium tuberculosis and Related Drug Resistance

## Supplemental material

- Supplemental file 1 -

  Tables S1 (Distribution of *M. tuberculosis* lineages by region of origin based on MIRU-VNTR and WGS typing), S2 (Patterns of resistance of *M. tuberculosis* strains to first-line anti-TB drugs based on phenotypic DST), S3 (Patterns of resistance of *M. tuberculosis* strains to second-line drugs based on phenotypic DST), S4 (Frequency of gene mutations conferring resistance to FLD compared with phenotypic DST results), and S5 (Distribution of gene mutations conferring resistance to SLD compared with phenotypic DST results)

  PDF, 284K
