# Supplementary material for: FIND Tuberculosis Strain Bank: a Resource for Researchers and Developers Working on Tests To Detect Mycobacterium tuberculosis and Related Drug Resistance
Source: J Clin Microbiol. 2017 Mar 24;55(4):1066–73. doi: 10.1128/JCM.01662-16 (PMC5377833; doi:10.1128/JCM.01662-16)
Supplement: Supplemental material [file JCM.01662-16_zjm999095424s1.pdf]

Table S1: Distribution of *M. tuberculosis* lineages by region of origin based on MIRU-VNTR and WGS typing (N=118)

M. tuberculosis lineages	Region of origin						Total	
	**Southeast Asia**		**Eastern Europe**		**South America**			
	**N**	**%**	**N**	**%**	**N**	**%**	**N**	**%**
**Beijing**	55	94.8	12	75.0	1	2.3	68	57.6
**LAM**	0	0.0	0	0.0	29	65.9	29	24.6
**X-type**	0	0.0	0	0.0	7	15.9	7	5.9
**URAL**	1	1.7	4	25.0	0	0.0	5	4.2
**Haarlem**	0	0.0	0	0.0	4	9.1	4	3.4
**Clade1**	1	1.7	0	0.0	3	6.8	4	3.4
**Delhi/CAS**	1	1.7	0	0.0	0	0.0	1	0.8
Total	58	100.0	16	100.0	44	100.0	118	100.0

N= number; LAM= Latin America-Mediterranean

Table S2: Resistance patterns of *M. tuberculosis* strains to first line anti-TB drugs based on phenotypic DST (N=118)

Patterns	First line drugs				Total	
	**INH**	**RMP#**	**EMB**	**PZA**	**N**	**%**
**1**	R	R	R	R	57	48.3
**2**	R	R	S	R	40	33.9
**3**	R	R	R	S	7	5.9
**4**	R	R	S	S	6	5.1
**5**	R	S	S	R	3	2.5
**6**	R	S	S	S	1	0.8
7	R	R	I	R	1	0.8
8	R	R	S	I	1	0.8
9	R	I	R	R	1	0.8
10	R	I	S	R	1	0.8
Total					118	100

N= number; R= resistant; S= susceptible; I = indeterminate; # = Four phenotypic RMP susceptible and two strains with indeterminate results were resistant to RMP based on the GeneXpert and sequencing results; INH= Isoniazid; RMP=Rifampicin; EMB = Ethambutol; PZA = Pyrazinamide

Table S3: Resistance patterns of *M. tuberculosis* strains to second-line drugs: FQ, SLIDs and ET based on phenotypic DST (N=113)

Resistance Patterns	Fluoroquinolones			Second line injectable drugs			ETH	Total	
	**OFL**	**MXF**	**LFX**	**AMK**	**CPM**	**KAN**	**ETH**	**N***	**%**
**1**	S	S	S	S	S	S	S	68	60.2
**2**	S	S	S	S	S	S	R	25	22.1
**3**	S	S	R	S	S	S	R	4	3.5
**4#**	R	R	R	R	R	R	R	2	1.8
**5**	S	S	S	R	S	S	S	2	1.8
**6**	S	S	R	R	R	R	R	2	1.8
**7**	S	S	S	R	R	S	S	1	0.9
**8**	S	S	S	R	R	R	R	1	0.9
**9**	S	S	R	S	S	S	S	1	0.9
**10**	S	S	R	R	R	R	S	1	0.9
**11**	S	R	S	S	S	S	R	1	0.9
**12**	R	S	R	S	S	S	S	1	0.9
**13**	R	S	R	S	S	S	R	1	0.9
**14**	R	R	R	S	S	S	S	1	0.9
**15**	R	R	R	S	S	S	R	1	0.9
Total								113	100.0

* = Five strains had indeterminate results for any of the SLDs tested; # = the two strains resistant to all second line drugs were also resistant to all first line drugs; OFL = Ofloxacin; MXF=Moxifloxacin; LFX = Levofloxacin; AMK = Amikacin; CPM = Capreomycin; KAM = Kanamycin; ETH = Ethionamide

Table S4: Frequency of gene mutations conferring resistance to FLD compared with phenotypic DST results

Anti-TB drugs	Genes	Mutations (Ref. 13)	Phenotypic DST			
			Frequency	R	S	I
		S450L	74	73	1	0
		D435V	12	12	0	0
		H445D	6	6	0	0
		S450W	3	3	0	0
RMP	*rpo*B	H445L	2	0	0	2
		Q432P	2	2	0	0
		L430P	1	0	1	0
		H445Y	1	1	0	0
		L452P	2	0	2	0
		H445R	2	2	0	0
		Q432K	1	1	0	0
		L452P	1	1	0	0
		H445D	1	1	0	0
		D435Y	1	1	0	0
		Unknown*	9	9	0	0
INH	*kat*G	S315T	102	102	0	0
		S315N	3	3	0	0
		W191R	1	1	0	0
	*inh*A	-15C/T	12	12	0	0
		M306I	28	13	14	1
		M306V	24	24	0	0
		Q497R	7	5	2	0
	*emb*B	Y319S	6	6	0	0
		G406A	4	1	3	0
		G406D	3	2	1	0
EMB		Y334H	3	3	0	0
		M306L	2	1	1	0
		Q497P	2	0	2	0
		F330S	1	1	0	0
		G416S	1	1	0	0

		M306D	1	1	0	0
		G406A	1	1	0	0
		V981L	1	0	1	0
		G406C	1	1	0	0
		V981L	1	0	1	0
	*emb*A	-11C/A	1	1	0	0
		-12C/T	1	1	0	0
		-16C/T	1	0	1	0
	*enb*B & *emb*A	-16C/T, S297A	2	2	0	0
		-8C/T, G406D	1	1	0	0
PZA	*pnc*A	-11A/G	7	7	0	0
		H51R	7	7	0	0
		Q10R	7	7	0	0
		DELETION	6	6	0	0
		Q10P	5	5	0	0
		A146V	4	4	0	0
		391insGG	4	4	0	0
		T47A	4	1	3	0
		D12A	3	3	0	0
		D12A	3	3	0	0
		H71R	3	3	0	0
		V139A	2	2	0	0
		W68G	2	2	0	0
		M175V	2	2	0	0
		-12T/G	1	1	0	0
		382insGG	1	1	0	0
		420insg	1	1	0	0
		A102V	1	0	1	0
		A146E	1	1	0	0
		A171E	1	1	0	0
		C138R	1	1	0	0
		C14Y	1	1	0	0
		D12G	1	1	0	0
		D136G	1	0	1	0

D56N, 164 DEL	1	1	0	0
D63G	2	2	0	0
Q141P	2	2	0	0
T76P	2	2	0	0
F58L	1	1	0	0
G108R	1	1	0	0
H137R	1	1	0	0
H82D	1	0	1	0
H82R	1	1	0	0
I6T	1	1	0	0
K48T	1	0	1	0
L116P	1	1	0	0
L120P	1	1	0	0
P54L	1	1	0	0
P62L	1	0	0	1
S67P	1	1	0	0
T100P	1	1	0	0
T135P	1	1	0	0
T160K	1	1	0	0
T160P	1	1	0	0
V128G	1	1	0	0
Y103OCHER	1	1	0	0
Unknown*	21	19	2	0

R = Resistant; S = Susceptible; I = Indeterminate; * = unidentified mutations; INH= Isoniazid; RMP=Rifampicin; EMB = Ethambutol; PZA = Pyrazinamide.

Table S5: Distribution of gene mutations conferring resistance to SLD compared with phenotypic DST results

Anti-TB drugs	Genes	Mutations (Ref. 13)	Phenotypic DST			
			Frequency	R	S	I
		D94G	6	6	0	0
Fluoroquinolones	*gyr*A	D94A	4	4	0	0
		D94Y	2	2	0	0
		D89N	1	1	0	0
	*gyr*B	E501D	1	1	0	0
	rrs	1401A/G	3	3	0	0
		1484G/T	1	1	0	0
Second line injectable drugs		-12C/T	4	4	0	0
	eis	-37G/T	2	2	0	0
		-14C/T	1	1	0	0
		-37C/T	1	1	0	0
	*tly*A	insGG	1	1	0	0
		Unknown*	6	6	0	0
		-15C/T	18	17	1	0
		-12T/C	1	1	0	0
ETH	*eth*A	-8T/C	1	1	0	0
		-9G/A	1	1	0	0
		I21V	1	1	0	0
		L35R	1	1	0	0
		Unknown*	18	17	1	0

R = Resistant; S = Susceptible; I = Indeterminate; * = unidentified mutations; ETH = Ethionamide
